# Supplementary material for: A nanosystem targeting tissue inhibitor of metalloproteinase-1 for continuous spatiotemporal idiopathic pulmonary fibrosis therapy
Source: Nat Commun. 2026 Jan 19;17:1694. doi: 10.1038/s41467-026-68398-0 (PMC12909971; doi:10.1038/s41467-026-68398-0)
Supplement: Supplementary file 1 — Supplementary information [file 41467_2026_68398_MOESM1_ESM.pdf]

Supplementary Information

A Nanosystem Targeting Tissue Inhibitor of Metalloproteinase-1 for Continuous Spatiotemporal Idiopathic Pulmonary Fibrosis Therapy

Chuyu Li[1#], Guihong Lu[2#], Hanlin Chen[1], Chenguang Wang[1], Zhongjie Wang[1], Ruiqi Ming[1], Shujun Liu[1] and Lili Huang[1,3*]

Correspondence to: llhuang@bit.edu.cn

1 School of Medical Technology, Beijing Institute of Technology, Beijing, People's Republic of China

2 Center for Child Care and Mental Health (CCCMH), Shenzhen Children's Hospital, Shenzhen, People's Republic of China

3 Tangshan Research Institute, Beijing Institute of Technology, Tangshan, People's Republic of China

#These authors contributed equally: Chuyu Li, Guihong Lu.

*Corresponding author: Lili Huang. e-mail: llhuang@bit.edu.cn.

This PDF file includes:

Table. S1

Fig. S1 — S29

Supplementary Tables

Table S1. Demographic information of IPF and healthy control lung tissues.

No.	Diagnosis
Non-IPF	
1	Atypical adenomatous hyperplasia
2	Lung squamous cell carcinoma
3	Lung adenocarcinoma
4	Lung adenoma
5	Benign pulmonary nodules
6	Lung adenocarcinoma
7	Benign pulmonary nodules
8	Lung adenocarcinoma
9	Benign pulmonary nodules
10	Lung adenocarcinoma
IPF	
1	Idiopathic pulmonary fibrosis
2	Idiopathic pulmonary fibrosis
3	Idiopathic pulmonary fibrosis
4	Idiopathic pulmonary fibrosis
5	Idiopathic pulmonary fibrosis
6	Idiopathic pulmonary fibrosis
7	Idiopathic pulmonary fibrosis
8	Idiopathic pulmonary fibrosis

Supplementary Figures

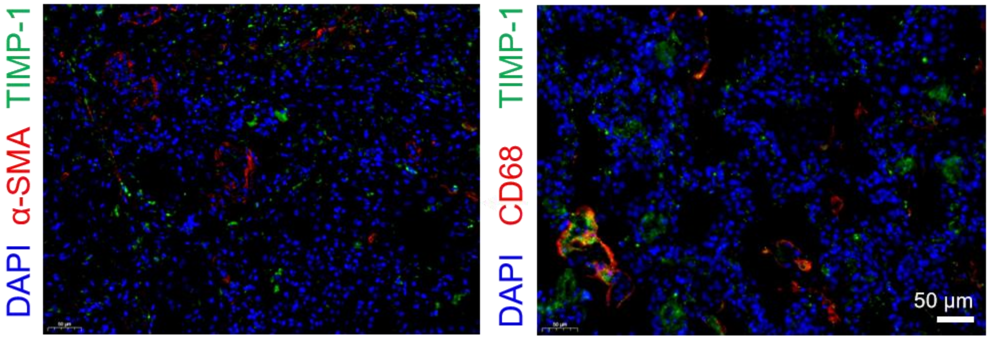

Fig. S1 IF staining of α-SMA, TIMP-1 and CD68 in human lung sections.

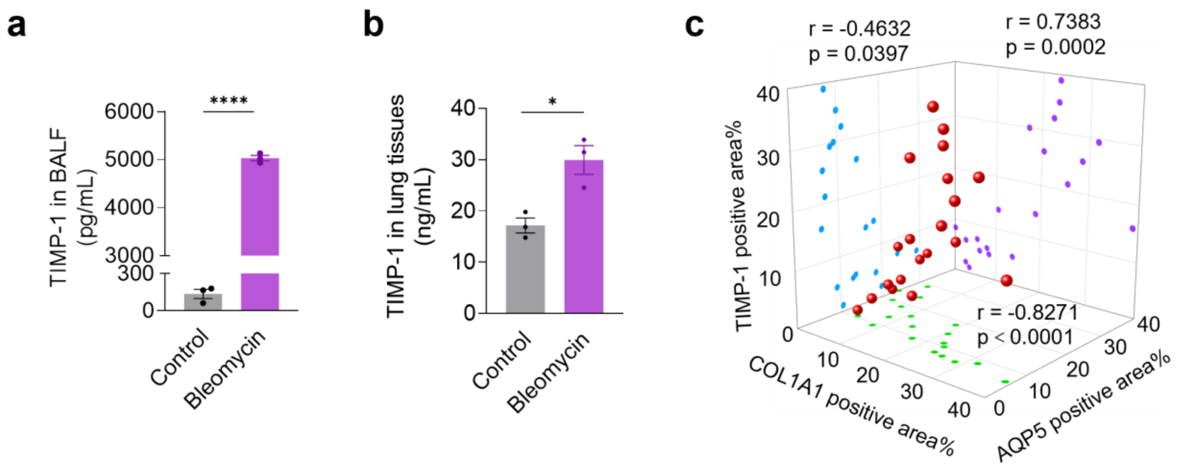

Fig. S2 TIMP-1 related analysis in a bleomycin-induced pulmonary fibrosis mouse model. a. The level of TIMP-1 in mouse BALF ($P < 0.0001$, n=3). **b.** The level of TIMP-1 in mouse lung tissues ($P = 0.0154$, n=3). **c.** Correlation of TIMP-1, COL1A1, and AQP5 in bleomycin-induced fibrosis mouse models (n=20). Correlation coefficient (r) was assessed using a nonparametric Spearman correlation analysis. Data are means ± SEM; *$P < 0.05$; ****$P < 0.0001$ (two-tailed unpaired Student's t test).

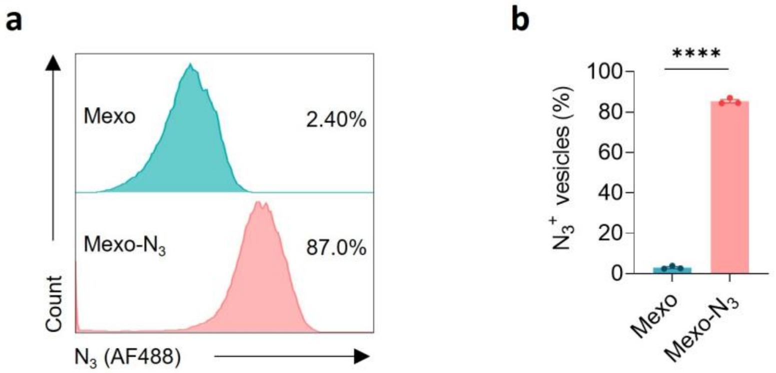

Fig. S3 Characterization of Mexo-N_3. a. Flow nanoanalysis of Mexo-N_3. N_3 was labeled with AF488-DBCO. **b.** Quantification of (a) ($P < 0.0001$, n=3). Data are means ± SEM; ****$P < 0.0001$ (two-tailed unpaired Student's *t* test).

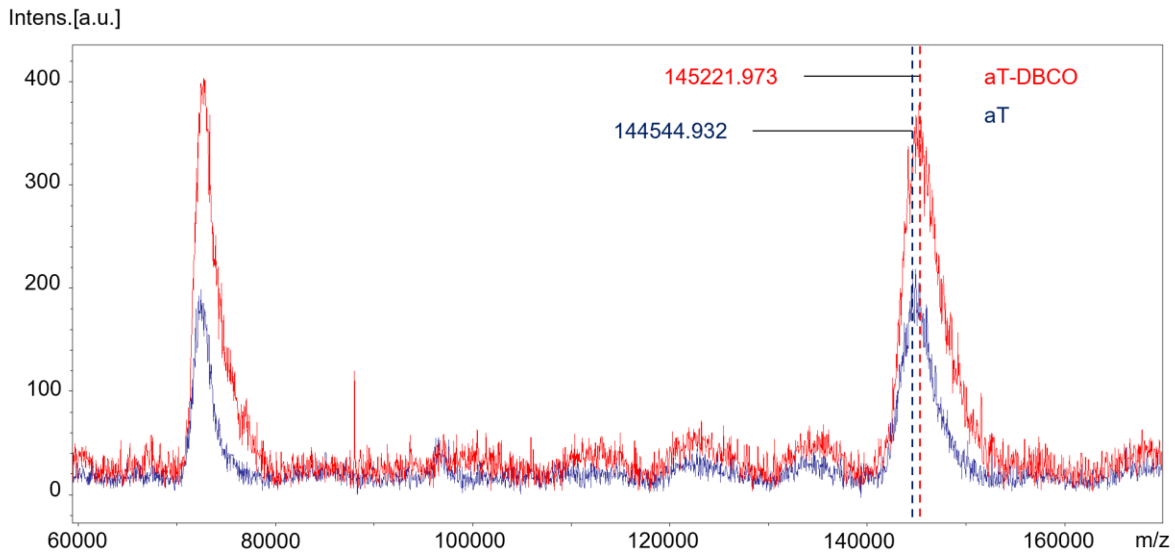

Fig. S4 Molecular weight analysis of aT-DBCO and aT by MALDI-TOF. The molecular weight increase of approximate 677 indicates that each aT is modified with an average of 1 to 2 DBCO molecules. The molecular weight of NHS-PEG$_4$-DBCO is 650.

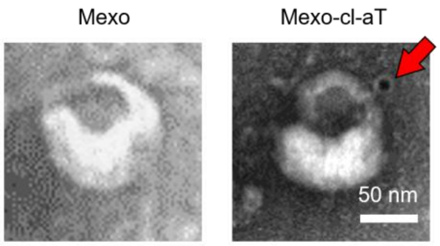

Fig. S5 TEM images of immunogold staining of aT. Red arrow indicates nanogold-labeled aT.

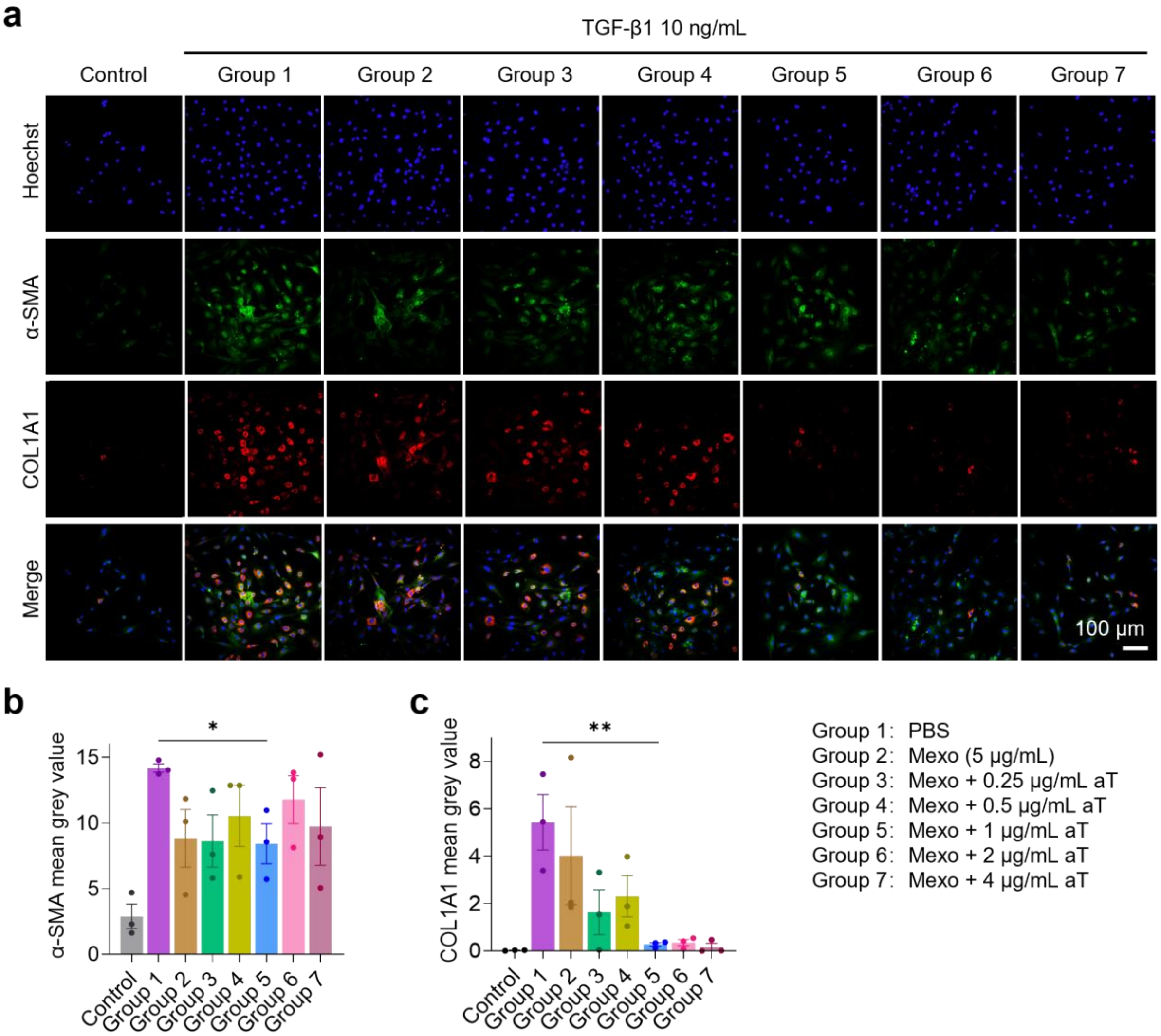

Fig. S6 Anti-fibrotic effects of various Mexo:aT ratio. a. IF staining of α-SMA and COL1A1 in MLFs. **b.** Quantification of α-SMA mean grey value (P=0.0497, n=3). **c.** Quantification of COL1A1 mean grey value (P=0.0016, n=3). Data are means ± SEM; ns, not significant; *P < 0.05; **P < 0.01 (one-way ANOVA with Sidak's multiple comparisons test).

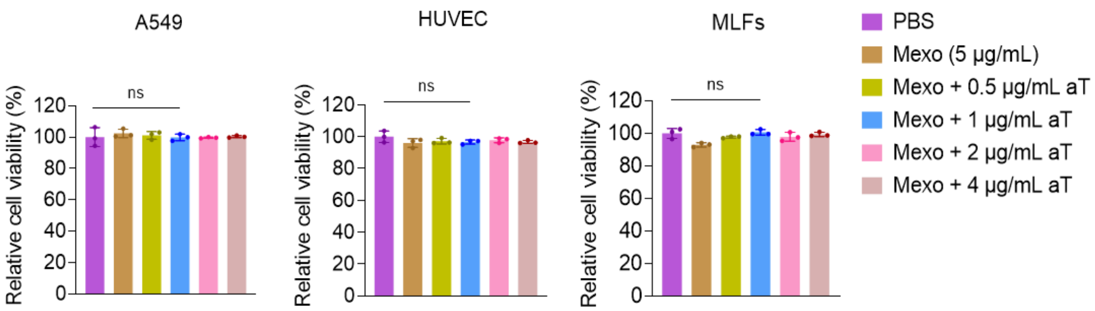

Fig. S7 The cytotoxic effect of Mexo-cl-aT on A549, HUVEC, and MLF cells by CCK8 assay. The cells were treated with different ratio of Mexo and aT for 24 hours before measurement (n=3). Data are means ± SEM; ns, not significant (one-way ANOVA with Sidak's multiple comparisons test).

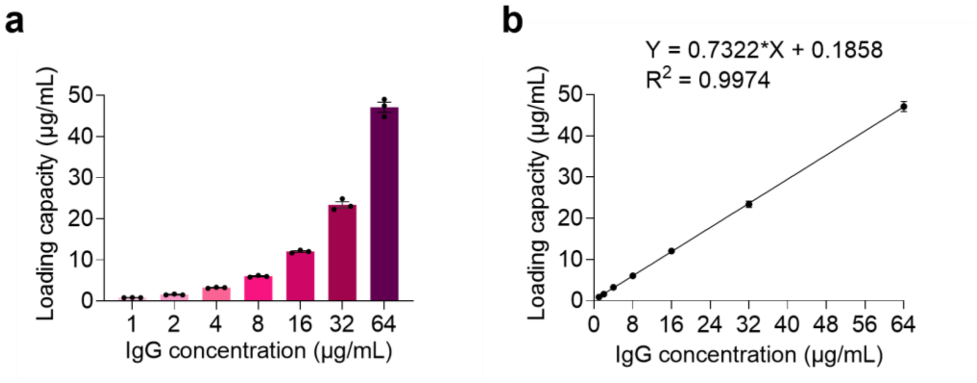

Fig. S8 Loading capacity of aT on Mexo. a. Quantification of aT on 1 mg/mL Mexo. **b.** Standard curve of loaded aT: initial aT by linear regression analysis (n=3). Data are means ± SEM.

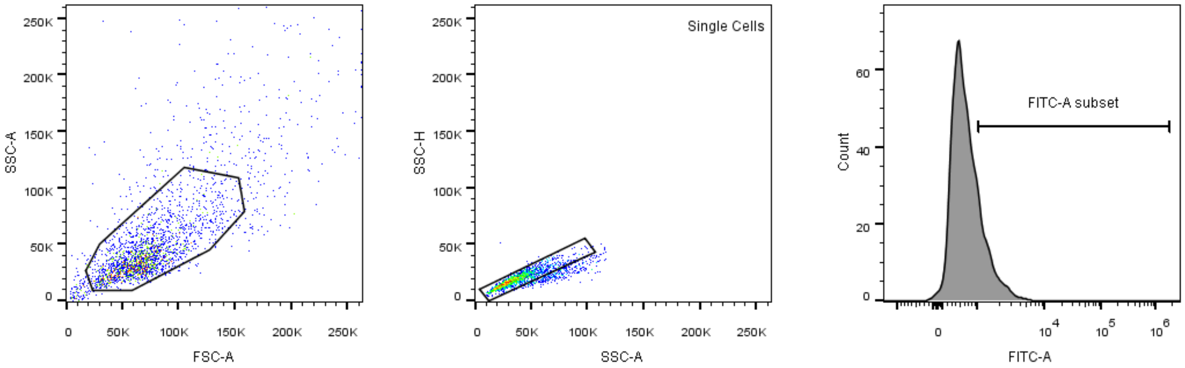

Fig. S9 Flow cytometry gating strategy corresponding to the flow cytometry analysis in Fig. 4b–c.

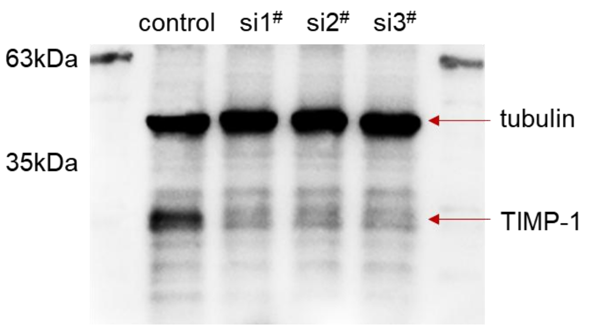

Fig. S10 Western blotting of TIMP-1 in MLFs after knockdown with different siRNAs.

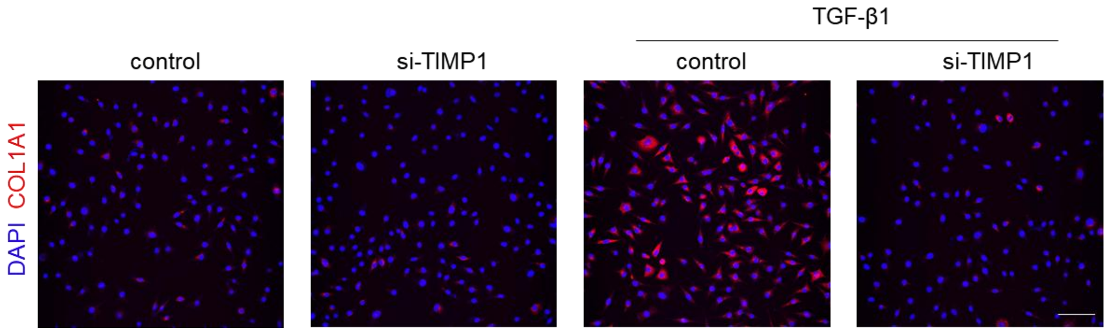

Fig. S11 Immunofluorescence staining of COL1A1 in control and TIMP-1-silenced MLFs, with or without TGF-β1 treatment. Scale bar = 100 μm.

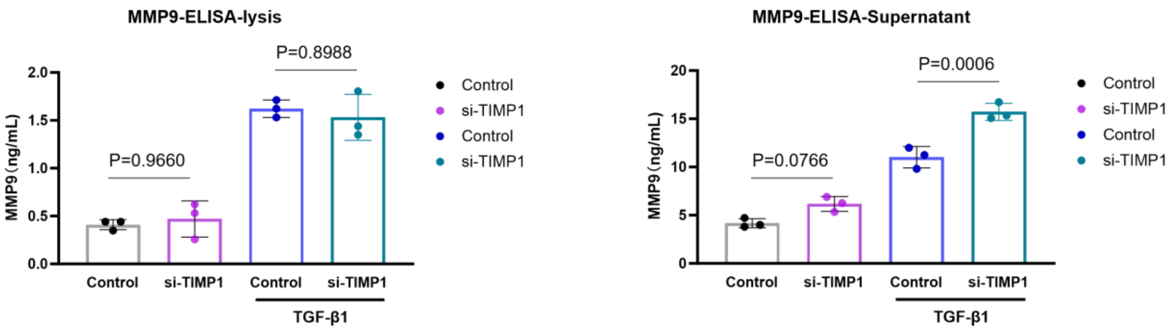

Fig. S12 Quantitative analysis of the active MMP-9 levels in cell lysates and supernatant of bleomycin-treated MLFs. PBS vs. TIMP-1-silenced MLFs; n=3. Data are means ± SEM; ne-way ANOVA with Dunnett's multiple comparisons test).

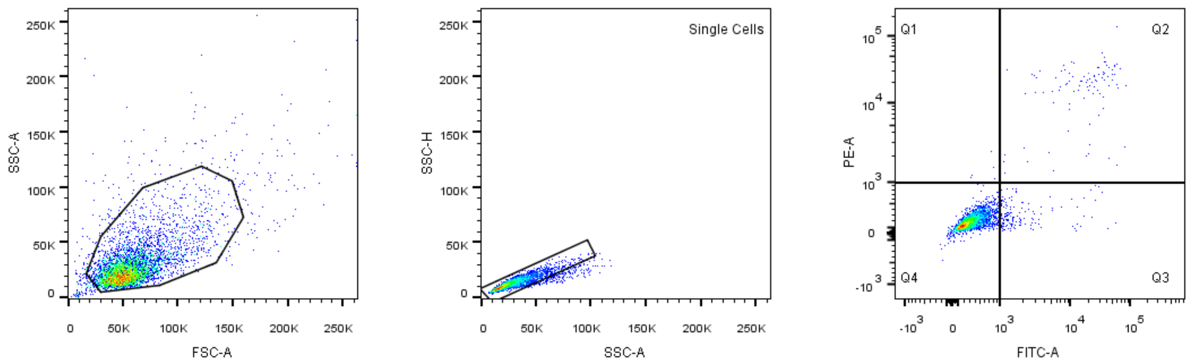

Fig. S13 Flow cytometry gating strategy corresponding to the flow cytometry analysis in Fig. 5d.

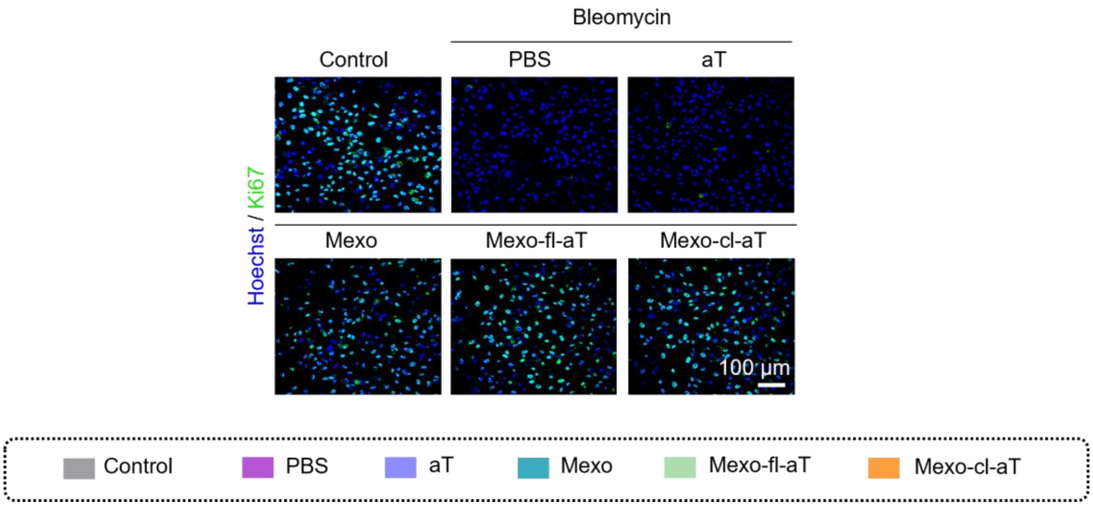

Fig. S14 Alveolar epithelial repair *in vitro*. CLSM images of A549 cells with Ki67 staining. Scale bar = 100 μm.

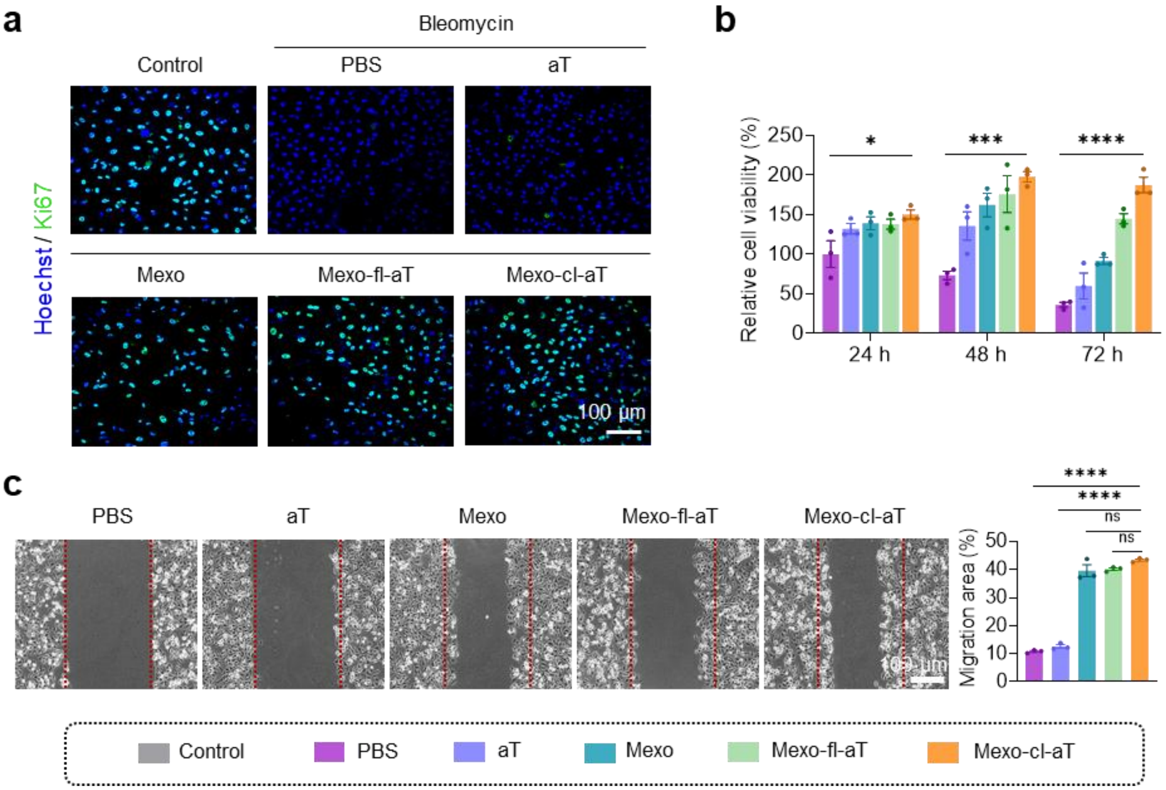

Fig. S15 HUVEC repair *in vitro*. a. CLSM images of HUVECs with Ki67 staining. Scale bar = 100 μm. **b.** Relative cell viability of HUVECs after various treatments for 24 ($P = 0.0143$), 48 ($P = 0.0006$), and 72 ($P < 0.0001$) hours (n=3). **c.** Images of wound healing assay and the quantification of migration area. (PBS vs. Mexo-cl-aT, $P < 0.0001$; n=3). Data are means ± SEM; ns, not significant; *$P < 0.05$; ***$P < 0.001$; ****$P < 0.0001$ (one-way ANOVA with Dunnett's multiple comparisons test).

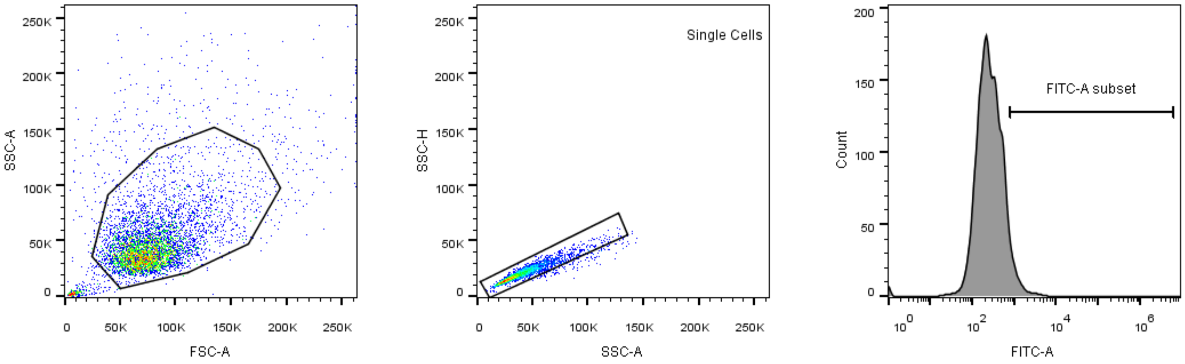

Fig. S16 Flow cytometry gating strategy corresponding to the flow cytometry analysis in Fig. 5h.

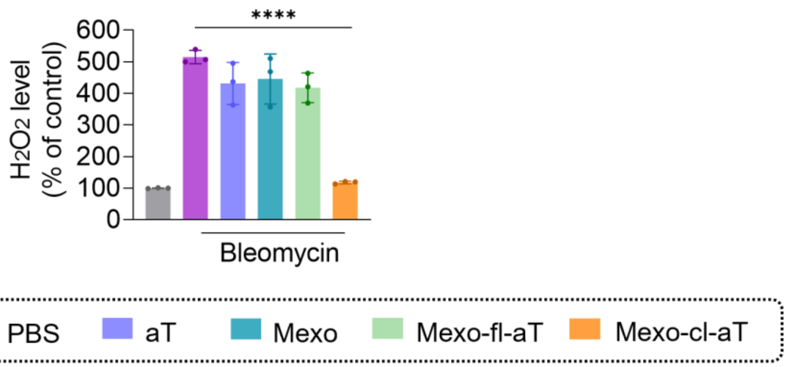

Fig. S17 Quantitative analysis of the H_2O_2 levels in the supernatant of bleomycin-treated MLFs. PBS vs. Mexo-cl-aT, $P < 0.0001$; n=3. Data are means ± SEM; ns, not significant; ****$P < 0.0001$ (one-way ANOVA with Dunnett's multiple comparisons test).

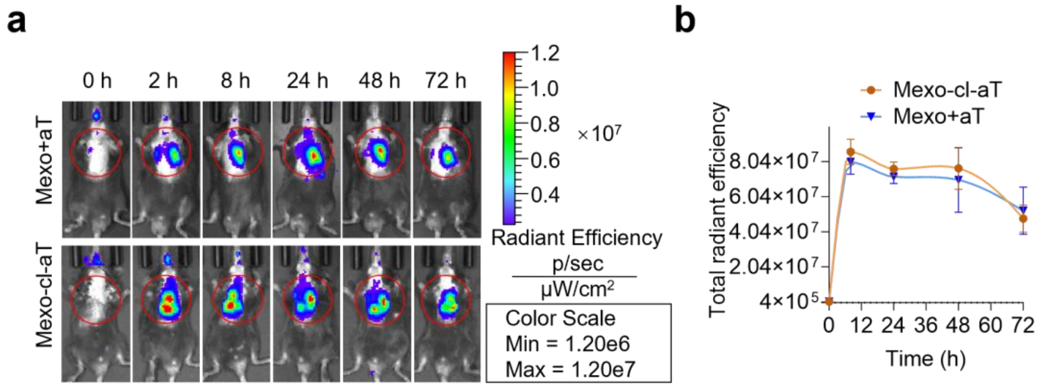

Fig. S18 Metabolism of Mexo-cl-aT in mouse fibrotic lungs. a. *In vivo* imaging of Mexo (DiR) signals in pulmonary fibrosis mouse model treated with Mexo+aT or Mexo-cl-aT. **b.** Total radiant efficiency curve of Mexo (DiR) (n=3). Data are means ± SEM.

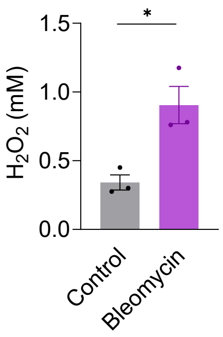

Fig. S19 The level of H_2O_2 in lung tissues of bleomycin-induced pulmonary fibrosis mouse model. P = 0.0181, n=3. Data are means ± SEM. *P < 0.05 (two-tailed unpaired Student's *t* test).

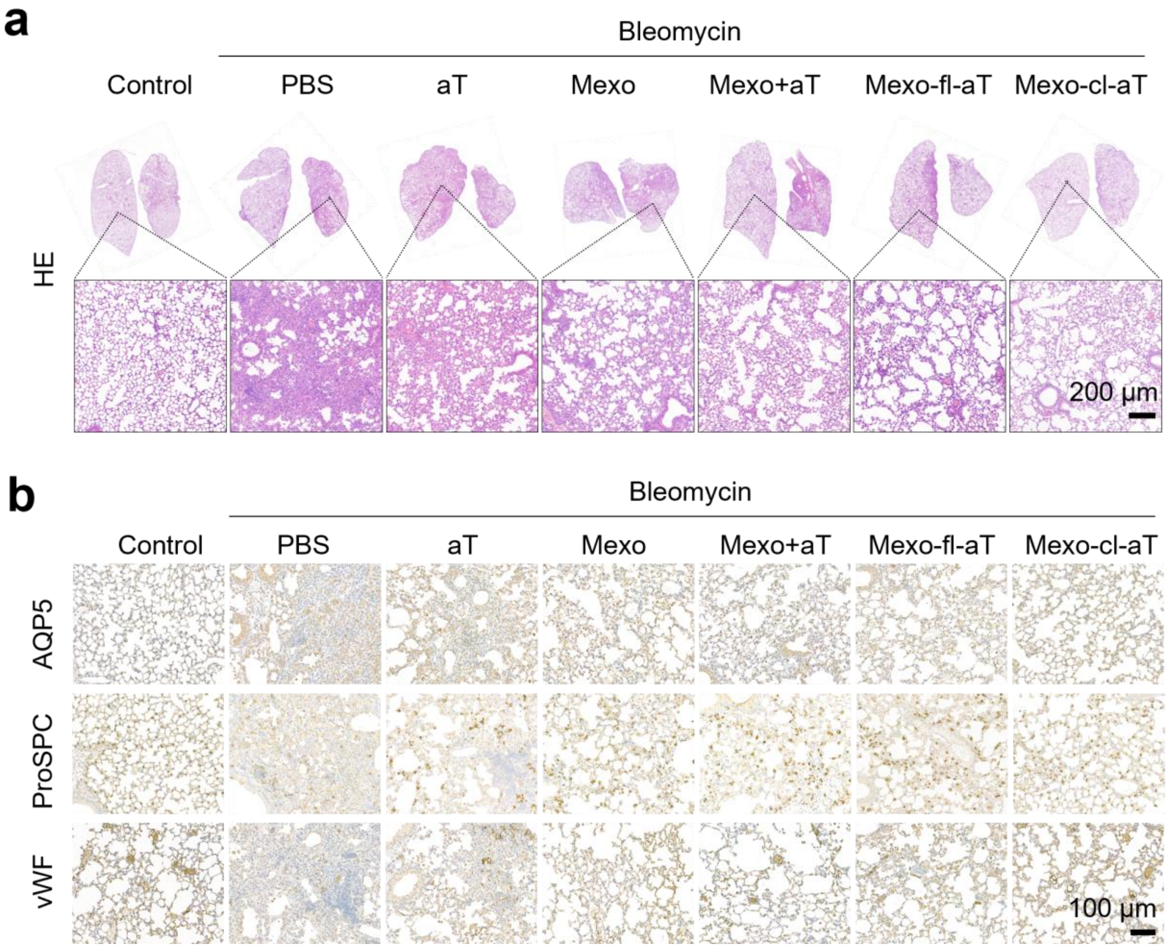

Fig. S20 Therapeutic effect of Mexo-cl-aT *in vivo*. a. HE staining of mouse lung sections. Scale bar = 200 μm. **b.** IHC staining of AQP5, ProSPC and vWF in mouse lung sections. Scale bar = 100 μm.

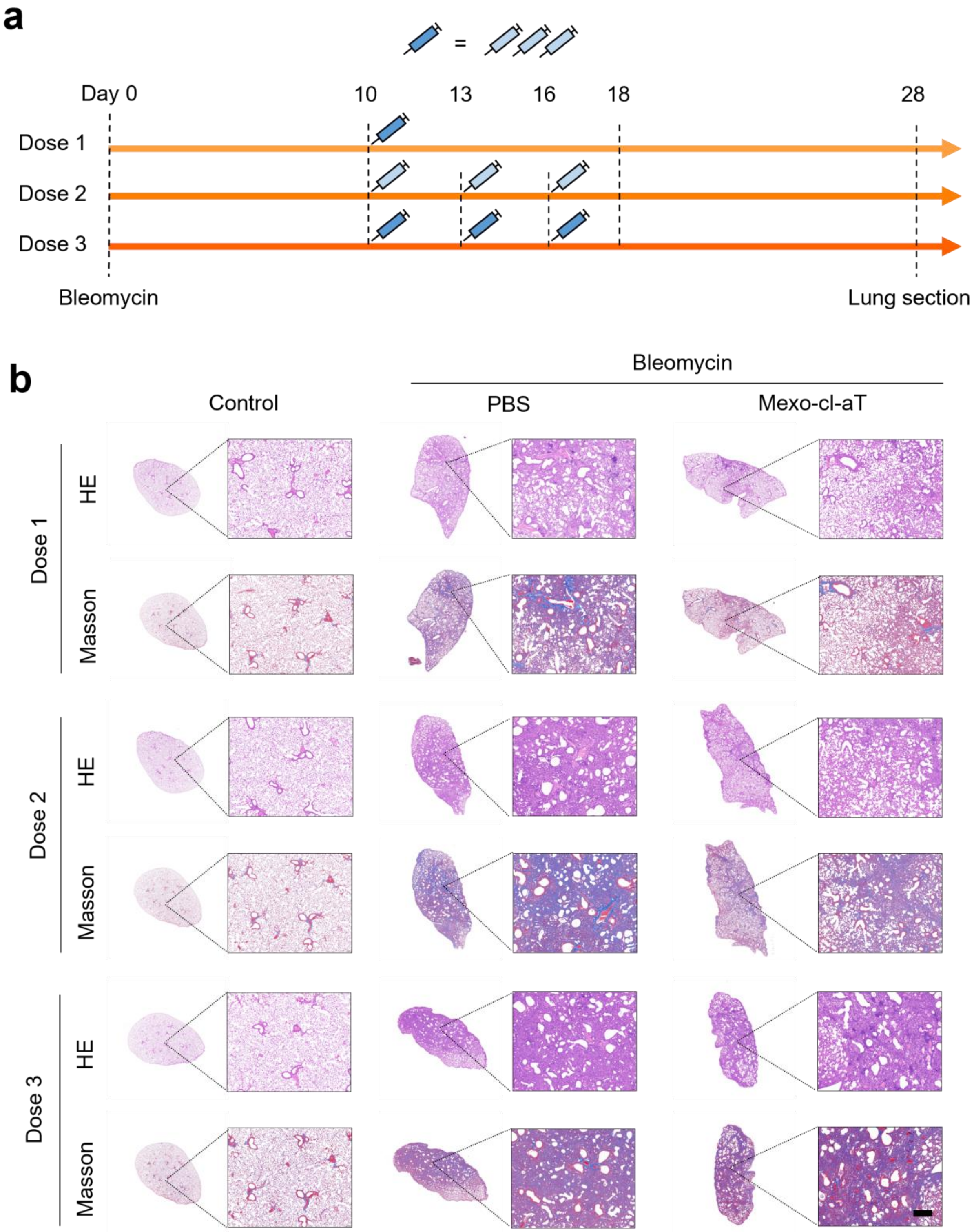

Fig. S21 Anti-fibrosis effect of different doses of Mexo-cl-aT. a. Illustration of animal experiment procedure. Created in BioRender. Zhang, F. (2025) https://BioRender.com/vwh725g.

b. H&E and Masson staining of mouse lungs after different treatments (n=6). Dose 1, high-dose single administration of Mexo-cl-aT (total equivalent: 10 μg Mexo and 2 μg aT). Dose 2, low-dose multiple administrations of Mexo-cl-aT (total equivalent: 10 μg Mexo and 2 μg aT). Dose 3, high-dose multiple administrations of Mexo-cl-aT (total equivalent: 30 μg Mexo and 6 μg aT). Scale bar = 200 μm.

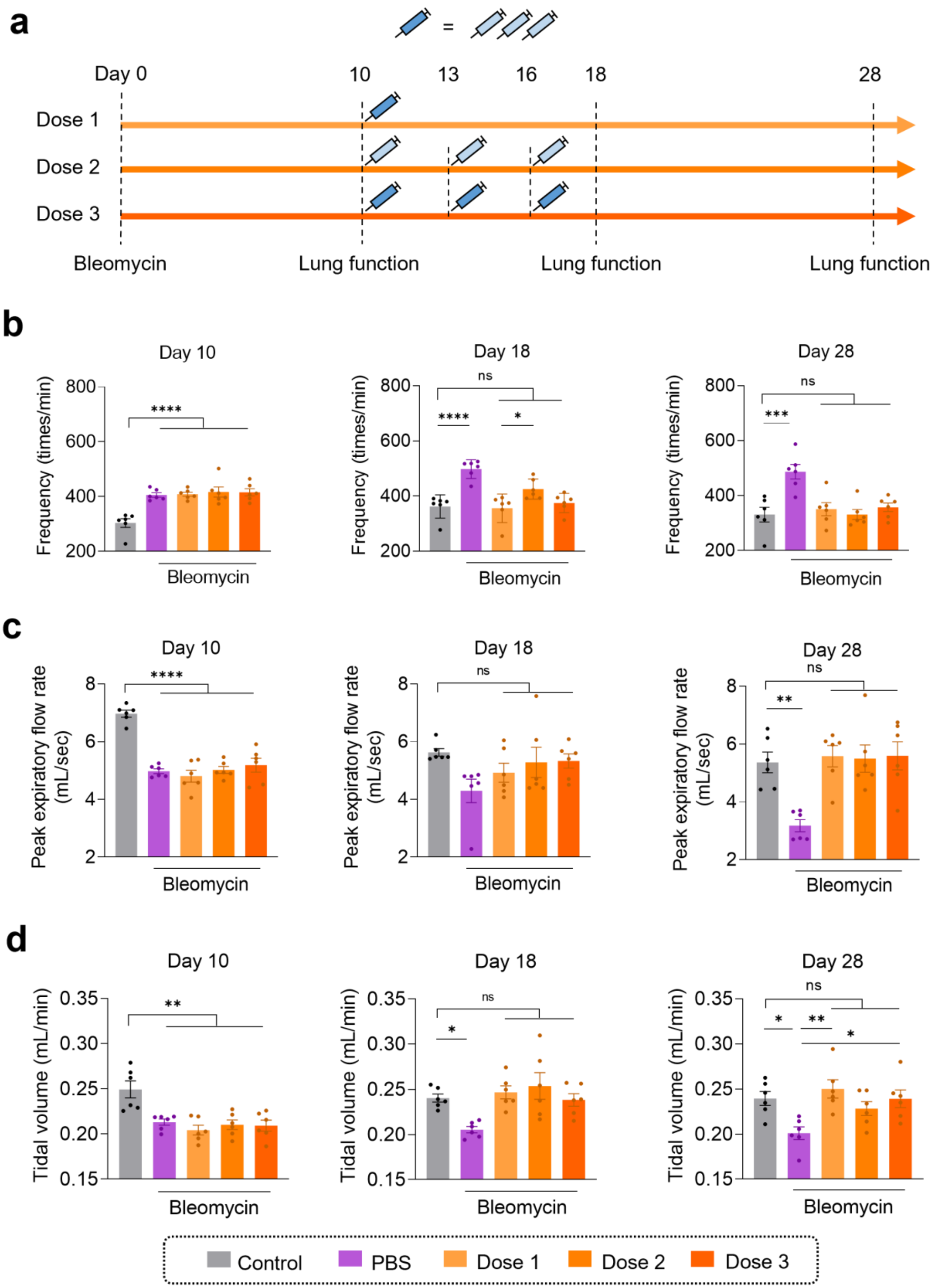

a
Day 0
10
13
16
18
28
Dose 1
Dose 2
Dose 3
Bleomycin
Lung function
Lung function
Lung function
b
Day 10
Day 18
Day 28
Frequency (times/min)
Bleomycin
c
Peak expiratory flow rate (mL/sec)
d
Tidal volume (mL/min)
Control
PBS
Dose 1
Dose 2
Dose 3

Fig. S22 Inhalation of Mexo-cl-aT restores the pulmonary function in mice of bleomycin-induced pulmonary fibrosis. a. Illustration of animal experiment procedure. Created in BioRender. Zhang, F. (2025) https://BioRender.com/b2w1zng. **b-d.** Measurements of pulmonary function parameters including frequency, (b), peak expiratory flow rate (c), and tidal volume (d) at day 10, 18, 28 treatment (n=6). Data are means ± SEM. Dose 1, high-dose single administration of Mexo-cl-aT (total equivalent: 10 μg Mexo and 2 μg aT). Dose 2, low-dose multiple administrations of Mexo-cl-aT (total equivalent: 10 μg Mexo and 2 μg aT). Dose 3, high-dose multiple administrations of Mexo-cl-aT (total equivalent: 30 μg Mexo and 6 μg aT). ns, not significant; *P < 0.05; **P < 0.01; ***P < 0.001; ****P < 0.0001 (one-way ANOVA with Dunnett's multiple comparisons test).

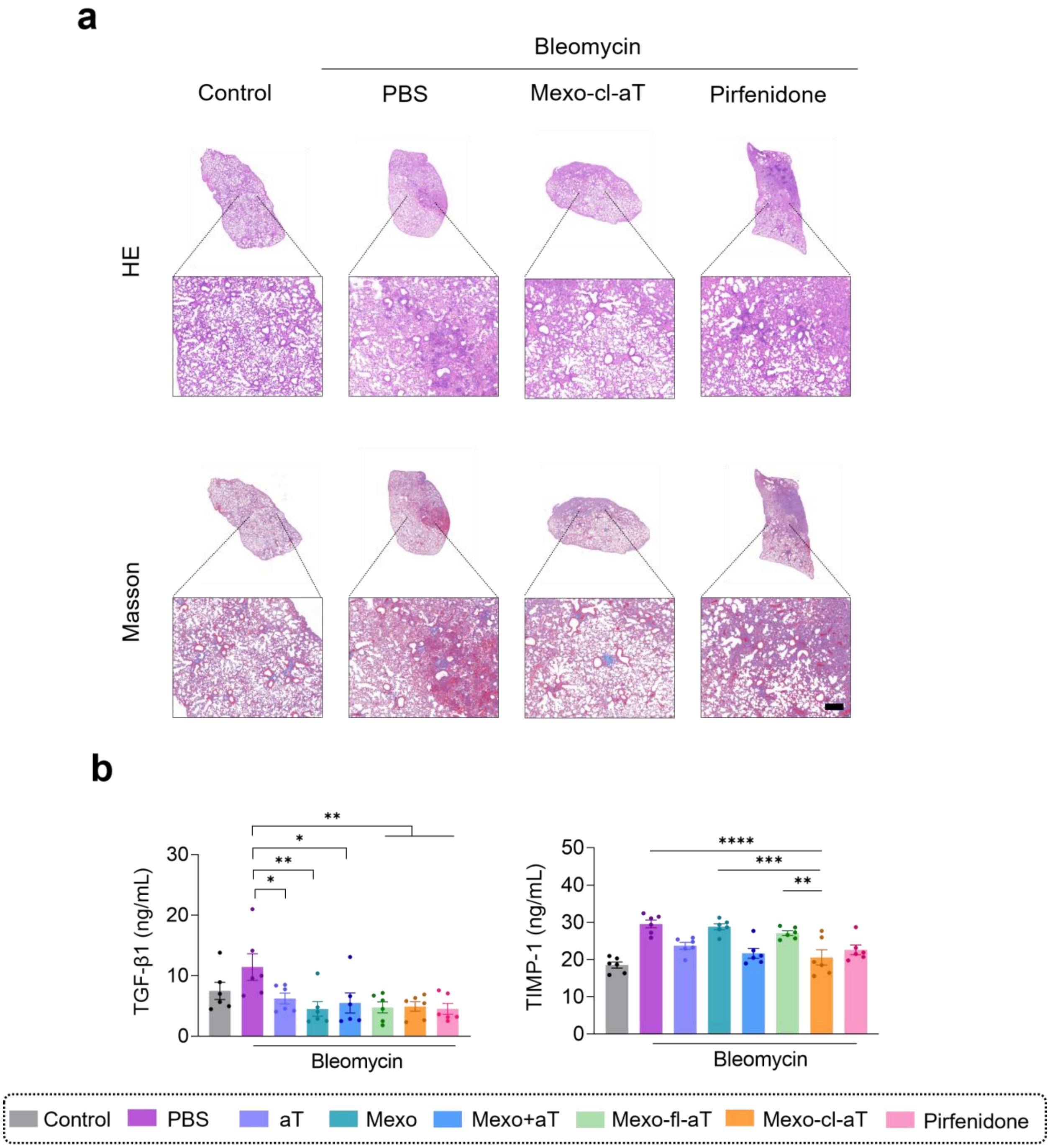

Fig. S23 Anti-fibrosis effect of Mexo-cl-aT *vs.* pirfenidone in a bleomycin-induced pulmonary fibrosis mouse model. a. HE and masson staining of the lung sections of mice treated with different formulations. Scale bar = 200 μm. **b.** The levels of TGF-β1 (PBS vs. Mexo-cl-aT, $P = 0.0076$; n=6) and TIMP-1 (PBS vs. Mexo-cl-aT, $P < 0.0001$; n=6) in mouse lung tissues determined

by ELISA. Data are means ± SEM. ns, not significant; *P < 0.05; **P < 0.01; ***P < 0.001; ****P < 0.0001 (one-way ANOVA with Dunnett's multiple comparisons test).

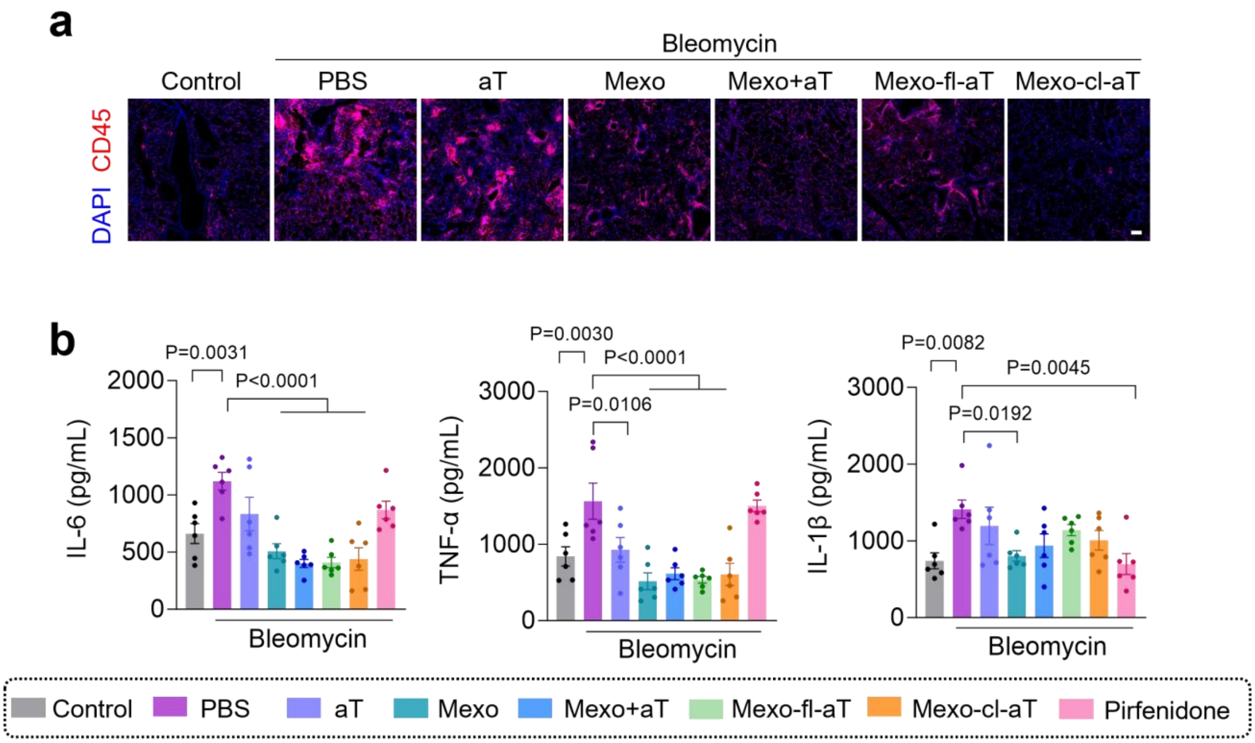

Fig. S24 Anti-inflammation of Mexo-cl-aT. a. Immunofluorescence staining of $CD45^+$ cells in mouse lung sections. Scale bar = 200 μm. **b.** The levels of IL-6 (PBS vs. Mexo-cl-aT, P < 0.0001; n=6), TNF-α (PBS vs. Mexo-cl-aT, P < 0.0001; N=6), and IL-1β (PBS vs. Mexo-cl-aT, P = 0.2009; n=6) in mouse lung tissues determined by ELISA. Data are means ± SEM. ns, not significant; *P < 0.05; **P < 0.01; ****P < 0.0001 (one-way ANOVA with Dunnett's multiple comparisons test).

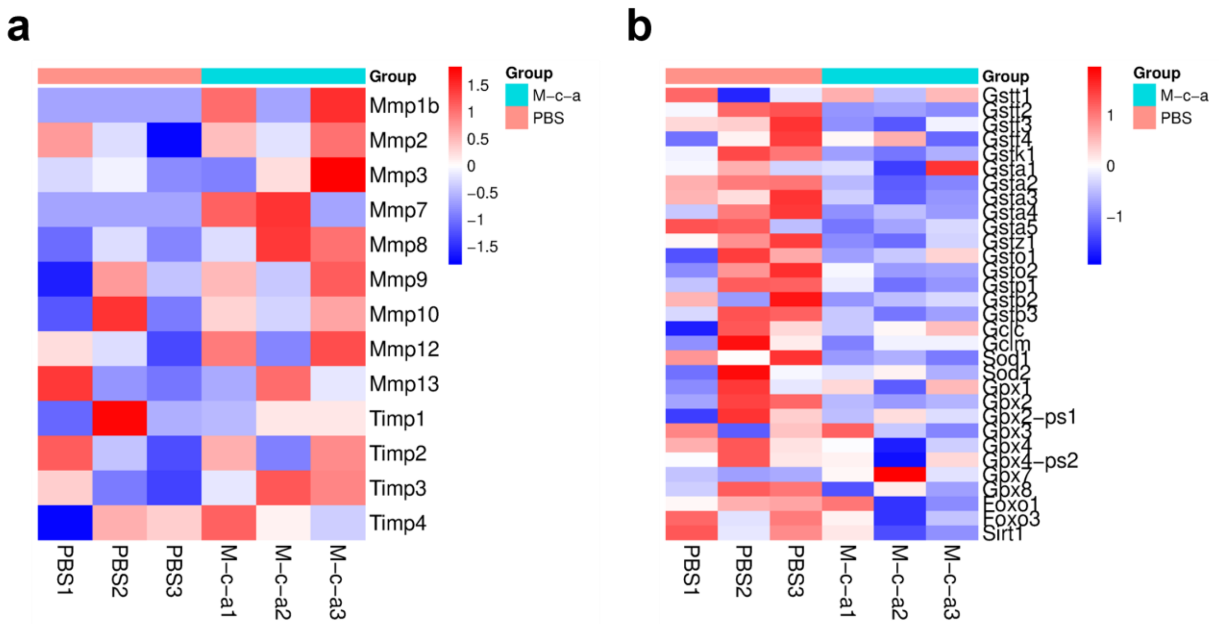

Fig. S25 Transcriptome analysis in the lung of mice with bleomycin-induced pulmonary fibrosis. a. Heatmap of MMP- and TIMP-related genes. **b.** Heatmap of oxidative stress-related genes. n=3. M-c-a: Mexo-cl-aT.

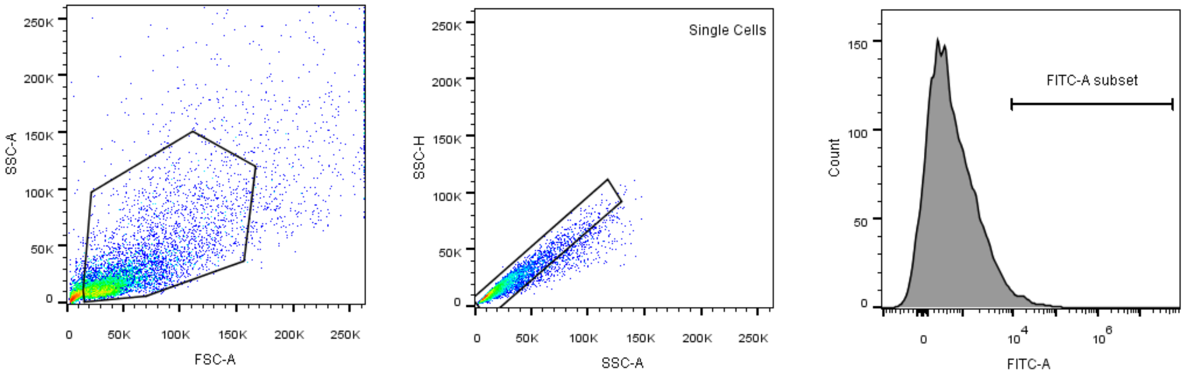

Fig. S26 Flow cytometry gating strategy corresponding to the flow cytometry analysis in Fig. 7i.

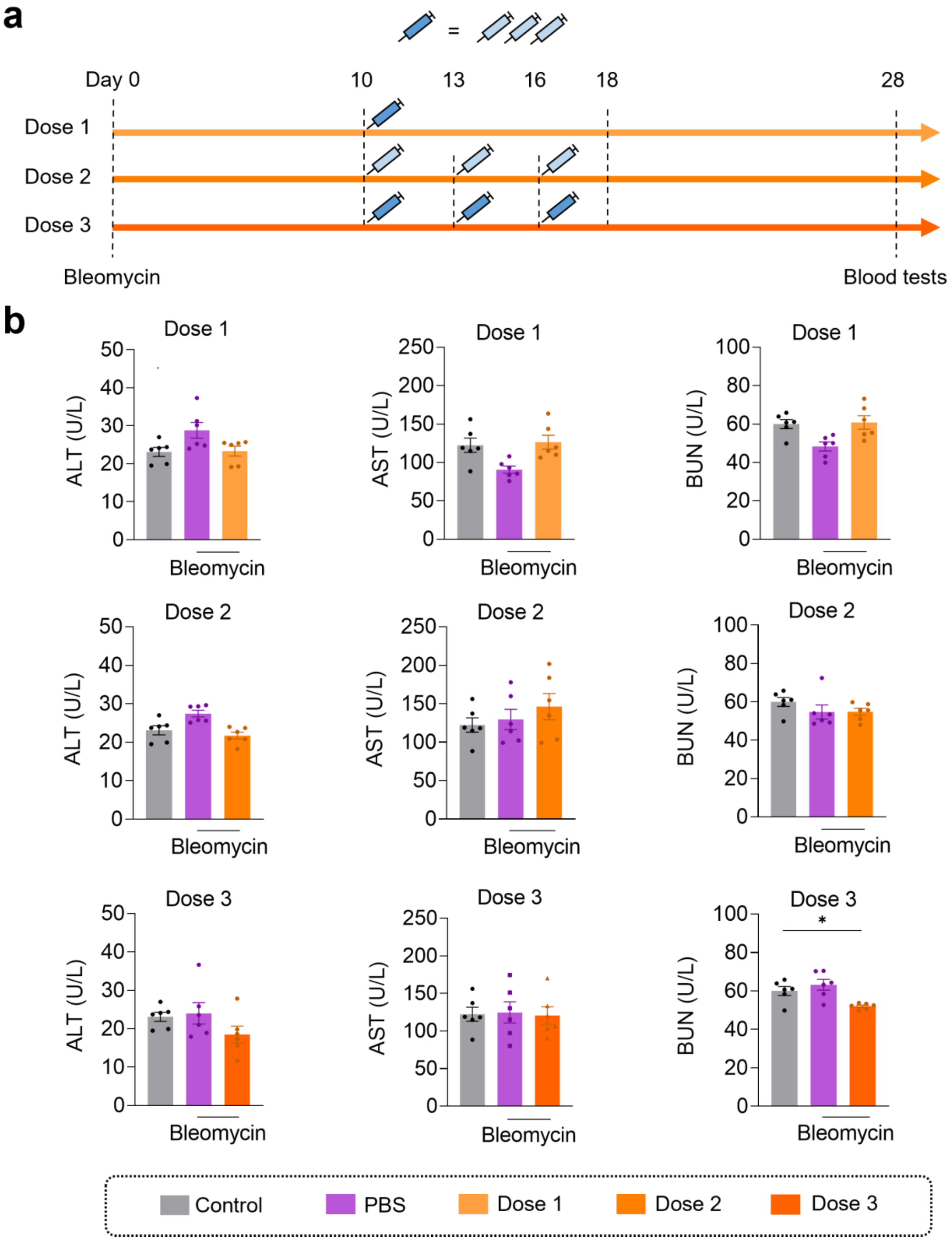

Fig. S27 Biosafety of different doses of Mexo-cl-aT treatment in a bleomycin-induced pulmonary fibrosis mouse model. a. Illustration of animal experiment procedure. Created in

BioRender. Zhang, F. (2025) https://BioRender.com/cfudszf. **b.** Evaluation of liver and kidney functions in mice treated with different doses of Mexo-cl-aT (n=6). Dose 1, high-dose single administration of Mexo-cl-aT (total equivalent: 10 μg Mexo and 2 μg aT). Dose 2, low-dose multiple administrations of Mexo-cl-aT (total equivalent: 10 μg Mexo and 2 μg aT). Dose 3, high-dose multiple administrations of Mexo-cl-aT (total equivalent: 30 μg Mexo and 6 μg aT). ns, not significant; *$P < 0.05$ (one-way ANOVA with Dunnett's multiple comparisons test).

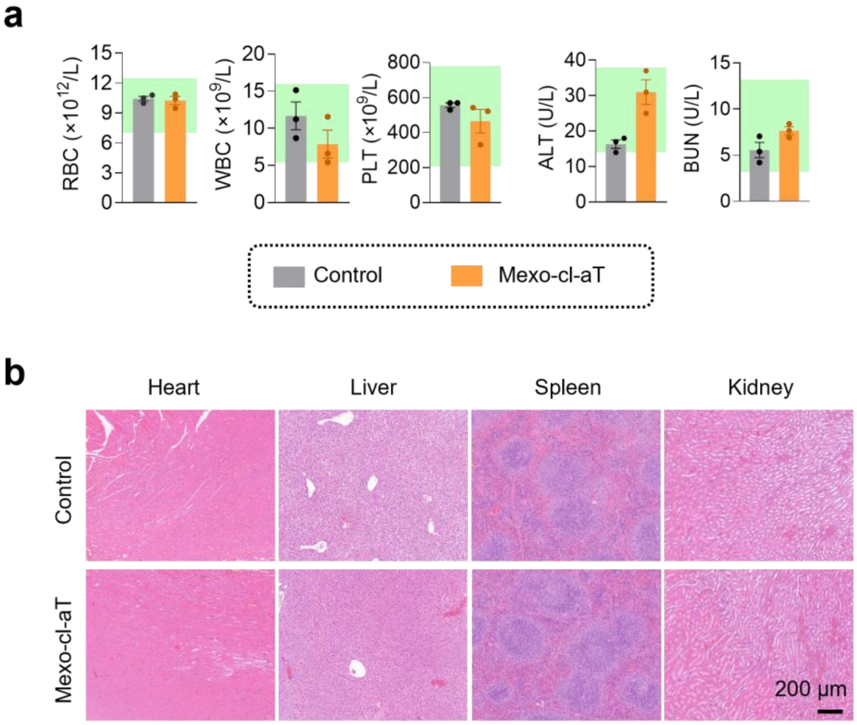

Fig. S28 Biosafety of Mexo-cl-aT. a. Hematologic and biochemical analysis of mice treated without/with Mexo-cl-aT (n=3). Green block indicates normal reference range. **b.** H&E staining of heart, liver, spleen and kidney sections. Data are means ± SEM.

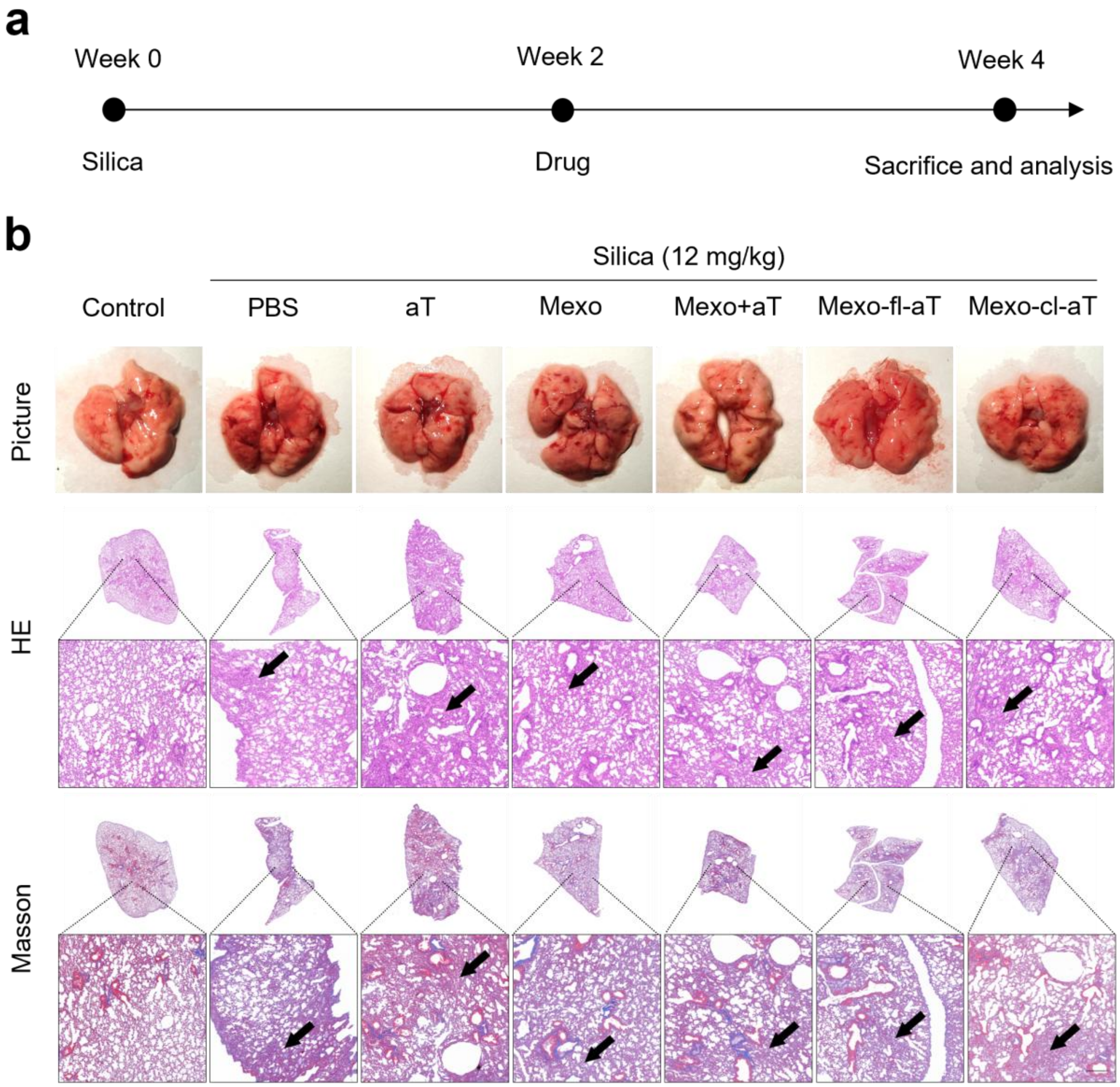

Fig. S29 Anti-fibrosis effect of Mexo-cl-aT in a silica-induced pulmonary fibrosis mouse model. a. Illustration of animal experiment procedure. **b.** Representative image, HE and masson staining of the lung sections of mice treated with different formulations. Arrows indicate fibrotic lesions. Scale bar = 200 μm.
