# Supplementary material for: A nanosystem targeting tissue inhibitor of metalloproteinase-1 for continuous spatiotemporal idiopathic pulmonary fibrosis therapy
Source: Nat Commun. 2026 Jan 19;17:1694. doi: 10.1038/s41467-026-68398-0 (PMC12909971; doi:10.1038/s41467-026-68398-0)
Supplement: Supplementary file 2 — Reporting Summary [file 41467_2026_68398_MOESM2_ESM.pdf]

nature portfolio

Corresponding author(s): Lili Huang

Last updated by author(s): Nov 11, 2025

Reporting Summary

Nature Portfolio wishes to improve the reproducibility of the work that we publish. This form provides structure for consistency and transparency in reporting. For further information on Nature Portfolio policies, see our Editorial Policies and the Editorial Policy Checklist.

Statistics

For all statistical analyses, confirm that the following items are present in the figure legend, table legend, main text, or Methods section.

n/a	Confirmed	
☐	☒	The exact sample size (*n*) for each experimental group/condition, given as a discrete number and unit of measurement
☐	☒	A statement on whether measurements were taken from distinct samples or whether the same sample was measured repeatedly
☐	☒	The statistical test(s) used AND whether they are one- or two-sided<br>*Only common tests should be described solely by name; describe more complex techniques in the Methods section.*
☒	☐	A description of all covariates tested
☒	☐	A description of any assumptions or corrections, such as tests of normality and adjustment for multiple comparisons
☐	☒	A full description of the statistical parameters including central tendency (e.g. means) or other basic estimates (e.g. regression coefficient) AND variation (e.g. standard deviation) or associated estimates of uncertainty (e.g. confidence intervals)
☐	☒	For null hypothesis testing, the test statistic (e.g. *F*, *t*, *r*) with confidence intervals, effect sizes, degrees of freedom and *P* value noted<br>*Give P values as exact values whenever suitable.*
☒	☐	For Bayesian analysis, information on the choice of priors and Markov chain Monte Carlo settings
☒	☐	For hierarchical and complex designs, identification of the appropriate level for tests and full reporting of outcomes
☐	☒	Estimates of effect sizes (e.g. Cohen's *d*, Pearson's *r*), indicating how they were calculated

Our web collection on statistics for biologists contains articles on many of the points above.

Software and code

Policy information about availability of computer code

Data collection	High-performance liquid chromatography (HPLC, Shimadzu, Japan) data were collected using LabSolutions LC software (ver1.25), Dynamic light scattering (DLS) and zeta potential data were collected using Zetasizer Software (ver7.11, Malvern Instruments, UK), Transmission electron microscopy (TEM) images were obtained using HITACHI TEM with TEM Imaging software, Western blot and SDS–PAGE chemiluminescence signals were collected using a gel imager (ThermoFisher, USA), and band intensities were quantified using ImageJ software, Confocal images were collected using CLSM (Nikon, Japan), Flow cytometry data were collected using BD FACSAriaII and further analyzed with FlowJo software (ver10.8), ELISA absorbance data were measured using a BioTek Synergy H1 microplate reader with Gen5 software (ver3.10), Cell viability data (CCK-8 assays) were measured using a microplate reader (Thermo Scientific, USA), Serum biochemical assay data (ALT, AST, ALP, LDH, BUN) were collected using Hitachi 7180 automated biochemical analyzer with LABOSPECT software, In vivo fluorescence and bioluminescence images were collected using IVIS Spectrum imaging system (PerkinElmer, USA) with Living Image software (ver4.5), Histological and immunohistochemical images were collected using Olympus SlideView VS200 Slide Scanner with Slideviewer software (ver2.6).
Data analysis	All statistical analysis was performed on Graphpad Prism 8. All imaging data were processed with Image J.

For manuscripts utilizing custom algorithms or software that are central to the research but not yet described in published literature, software must be made available to editors and reviewers. We strongly encourage code deposition in a community repository (e.g. GitHub). See the Nature Portfolio guidelines for submitting code & software for further information.

Data

Policy information about availability of data

All manuscripts must include a data availability statement. This statement should provide the following information, where applicable:

- Accession codes, unique identifiers, or web links for publicly available datasets
- A description of any restrictions on data availability
- For clinical datasets or third party data, please ensure that the statement adheres to our policy

Provide your data availability statement here.

Research involving human participants, their data, or biological material

Policy information about studies with human participants or human data. See also policy information about sex, gender (identity/presentation), and sexual orientation and race, ethnicity and racism.

Reporting on sex and gender	Clinical specimens used in our experiment were obtained from both male and female patients, and patient sex is indicated in Table 1. The experiment was designed without considering the sex of patient.
Reporting on race, ethnicity, or other socially relevant groupings	Not applicable.
Population characteristics	Patients aged 18 years or older with idiopathic pulmonary fibrosis (IPF) were enrolled in this study; those with comorbid chronic inflammatory diseases, including COPD or bronchiectasis, were excluded. All human donors provided informed consent.
Recruitment	Diseased lung tissue was obtained from patients with a diagnosis of idiopathic pulmonary fibrosis (IPF) based on standard diagnostic criteria and multi-disciplinary team (MDT) consensus, at the point of lung transplantation at the First Affiliated Hospital, Zhejiang University School of Medicine. Control lung samples from non-cancerous areas of lung cancer patients or non-diseases areas of benign nodule patients were also obtained for immunohistochemistry (IHC) and immunofluorescence (IF) staining.
Ethics oversight	Human specimens<br>Patient characteristics are detailed in Supplementary Table S1. Specimens were collected from the First Affiliated Hospital, Zhejiang University School of Medicine. All the procedures were approved by the Clinical Research Ethics Committee of First Affiliated Hospital, Zhejiang University School of Medicine (approval ID: 1047) and Shenzhen Children's Hospital (approval ID: 202315902).<br>Animals<br>All the procedures were approved by the Animal Ethics Association and the Ethics Committee of Beijing Institute of Technology (approval ID: 2022-0009-167).

Note that full information on the approval of the study protocol must also be provided in the manuscript.

Field-specific reporting

Please select the one below that is the best fit for your research. If you are not sure, read the appropriate sections before making your selection.

☒ Life sciences ☐ Behavioural & social sciences ☐ Ecological, evolutionary & environmental sciences

For a reference copy of the document with all sections, see nature.com/documents/nr-reporting-summary-flat.pdf

Life sciences study design

All studies must disclose on these points even when the disclosure is negative.

Sample size	No statistical methods were used to predetermine sample sizes. Sample sizes for animal experiments were determined based on preliminary pilot studies (Itahashi et al., Sci Immunol., 2022; Kumagai et al., Cancer Cell., 2022; Kumagai et al., Nat Immunol., 2020), and were in line with standards in the field (Tanoue et al., Nature., 2019; Mager et al., Science., 2020; Gopalakrishnan et al., Science., 2018).For in vivo bleomycin-induced IPF mouse models, 6–8 mice per group were used—referencing anti-fibrotic study standards, supported by a priori power analysi. In vitro cellular experiments (e.g., viability, ROS scavenging) included 3 biological replicates with 3–4 technical replicates each, following field norms to reduce variabilit, 3 independent batches were used per assay (based on lab protocols) to verify formulation stabilit.
Data exclusions	No data exclusions were needed.
Replication	All in vitro and in vivo experiments were conducted at least 3 times with similar results. Regarding reproducibility of the multiplex immunohistochemistry staining data, three independent regions of interest (ROIs: 940 µm × 1175 µm) were analyzed per tissue sample.

Randomization	No randomization was performed in the human study, as it was observational. In the animal experiments, mice were randomly assigned to different groups prior to challenge with bleomycin and/or intratracheal administration of Mexo-cl-aT.
Blinding	No blinded experiments were conducted in human study, because it was an observational study. All other animal studies were not blinded due to requirements for cage labeling and staffing logistics.

Reporting for specific materials, systems and methods

We require information from authors about some types of materials, experimental systems and methods used in many studies. Here, indicate whether each material, system or method listed is relevant to your study. If you are not sure if a list item applies to your research, read the appropriate section before selecting a response.

Materials & experimental systems

n/a	Involved in the study
☐	☒ Antibodies
☐	☒ Eukaryotic cell lines
☒	☐ Palaeontology and archaeology
☐	☒ Animals and other organisms
☐	☒ Clinical data
☒	☐ Dual use research of concern
☒	☐ Plants

Methods

n/a	Involved in the study
☒	☐ ChIP-seq
☐	☒ Flow cytometry
☒	☐ MRI-based neuroimaging

Antibodies

Antibodies used	Rabbit anti mouse COL1A1 antibody (Thermo, PA5-29569, 1:1000); Alexa Fluor 488-conjugated rabbit anti mouse α-SMA antibody (abcam, ab202295, 1:100); Alexa Fluor 647-conjugated goat anti rabbit IgG secondary antibody (Thermo, A27040, 1:200); Rabbit anti mouse AQP5 antibody (Thermo, PA5-36529,1:200); Rabbit anti mouse ProSPC antibody (abcam, ab90716, 1:200).
Validation	All antibodies used in this study are commercially available. Antibodies employed in flow cytometry, multiplex immunohistochemistry, confocal imaging, and immunohistochemistry were validated by the respective manufacturers for use in the relevant species and applications, as stated on the manufacturers' websites. Validation details can be found at the following URLs: Thermo Fisher (https://www.thermofisher.com/jp/ja/home/life-science/antibodies/invitrogen-antibody-validation.html), abcam (https://www.abcam.co.jp/primary-antibodies/how-we-validate-ourantibodies), All antibodies were used according to the manufacturers' instructions.

Eukaryotic cell lines

Policy information about cell lines and Sex and Gender in Research

Cell line source(s)	Human umbilical cord mesenchymal stem cells (HUMSCs, 4201PAT-CCTCC02079) were obtained from the Cell Resource Center, Peking Union Medical College, and cultured in complete HMSC medium (SIMPSONLIFE, China). A549 cells (CRM-CCL-185) and HUVECs (PCS-100-013) were obtained from ATCC and cultured in high glucose DMEM containing 10% FBS, penicillin (100 U/mL), and streptomycin (100 μg/mL) at 37°C under 5% CO2 environment. Primary MLFs were obtained from C57BL/6J mouse lung tissues. Briefly, the tissues were harvested and cut into small pieces (1 mm3), digested in type IV collagenase (2 mg/ml) at 37°C for 15-20 min, terminated and washed in high glucose DMEM containing 20% FBS and antibiotics. Small pieces were spread on the petri dish (diameter 10 cm) and cultured in 2 mL complete medium for 24 hours for stable adherence, followed by 4 mL medium replacement. Fibroblasts climbed out from the margin of small tissue on day 3. The fibroblasts were subcultured when the density reached 80%. Primary Mouse lung fibroblasts (MLFs) were harvested from male mice (C57BL/6), and they were used for downstream experiments between passages 3 and 5.
Authentication	No further authentication was performed for commercially available cell lines. The MLFs were stimulated with TGF-β1 and validated with immunofluorescence staining.
Mycoplasma contamination	All cell lines were regulatory tested and negative for mycoplasma contamination.
Commonly misidentified lines (See ICLAC register)	No commonly misidentified cell lines were used in the study.

Animals and other research organisms

Policy information about studies involving animals; ARRIVE guidelines recommended for reporting animal research, and Sex and Gender in Research

Laboratory animals	C57BL/6J male mice (8-10 weeks) were purchased from Laboratory Animal Center, Peking University.
Wild animals	No wild animals were used.

Reporting on sex	The experiment was designed without considering the sex of the mice, and male mice were selected to ensure gender uniformity
Field-collected samples	No field-collected samples were used.
Ethics oversight	All the procedures were approved by the Animal Ethics Association and the Ethics Committee of Beijing Institute of Technology (approval ID: 2022-0009-167) Zhejiang University School of Medicine (approval ID: 1047) and Shenzhen Children's Hospital (approval ID: 202315902).

Note that full information on the approval of the study protocol must also be provided in the manuscript.

Clinical data

Policy information about clinical studies
All manuscripts should comply with the ICMJE guidelines for publication of clinical research and a completed CONSORT checklist must be included with all submissions.

Clinical trial registration	This is not a clinical trial, but an observational study.
Study protocol	Not applicable.
Data collection	Clinical data were collected from medical records.
Outcomes	Not applicable.

Plants

Seed stocks	Not applicable.
Novel plant genotypes	Not applicable.
Authentication	Not applicable.

Flow Cytometry

Plots

Confirm that:

☒ The axis labels state the marker and fluorochrome used (e.g. CD4-FITC).

☒ The axis scales are clearly visible. Include numbers along axes only for bottom left plot of group (a 'group' is an analysis of identical markers).

☒ All plots are contour plots with outliers or pseudocolor plots.

☒ A numerical value for number of cells or percentage (with statistics) is provided.

Methodology

Sample preparation	To treated cells were collected from glass bottom dishes, filtered using a 70-µm strainer and stained with Ki67, COL1A1 primary antibodies, TRITC-conjugated secondary antibodies, and Alexa Fluor 488-conjugated α-SMA antibodies.
Instrument	BD FACSAriaII, USA
Software	Flow cytometry data were collected using BD FACSDiva software (ver8.0.3) and further analyzed with FlowJo software (ver10.8).
Cell population abundance	Post-sort purities were greater than 90%.
Gating strategy	Generally, cells were first gated based on FSC-A/SSC-A and FSC-A/FSC-H to select for live single cells.

☒ Tick this box to confirm that a figure exemplifying the gating strategy is provided in the Supplementary Information.
